# Biochemical and Biological Profile of Parotoid Secretion of the Amazonian* Rhinella marina* (Anura: Bufonidae)

**DOI:** 10.1155/2019/2492315

**Published:** 2019-02-21

**Authors:** Daniel S. S. de Medeiros, Tiago B. Rego, Ana P. de A. dos Santos, Adriana S. Pontes, Leandro S. Moreira-Dill, Najla B. Matos, Juliana P. Zuliani, Rodrigo G. Stábeli, Carolina B. G. Teles, Andreimar M. Soares, Angelo R. de M. Sperotto, Dinara J. Moura, Jenifer Saffi, Cleópatra Alves da Silva Caldeira, Daniel Carvalho Pimenta, Leonardo A. Calderon

**Affiliations:** ^1^Centro de Estudo de Biomoléculas Aplicadas à Saúde (CEBio), FIOCRUZ-Rondônia, Porto Velho, 76812-245, Brazil; ^2^Plataforma de Bioensaios de Malária e Leishmaniose (PBML), FIOCRUZ-Rondônia, Porto Velho, 76812-245, Brazil; ^3^Instituto Nacional de Epidemiologia na Amazônia Ocidental, Porto Velho, 76812-245, Brazil; ^4^Laboratório de Imunologia Aplicada à Saúde, FIOCRUZ-Rondônia, Porto Velho, 76812-245, Brazil; ^5^Laboratório de Microbiologia, FIOCRUZ-Rondônia, Porto Velho, 76812-245, Brazil; ^6^Laboratório de Genética Toxicológica, Universidade Federal de Ciências da Saúde de Porto Alegre, Porto Alegre, 90050-170, Brazil; ^7^Laboratório de Bioquímica e Biofísica, Instituto Butantan, Avenida Vital Brazil, 1500, 05503-900 Sao Paulo, SP, Brazil

## Abstract

Skin secretions of frogs have a high chemical complexity. They have diverse types of biomolecules, such as proteins, peptides, biogenic amines, and alkaloids. These compounds protect amphibians' skin against growth of bacteria, fungi, and protozoa and participate in defense system against attack from predators. Therewith, this work performed biochemical and biological profile of macroglands parotoid secretion from cane toad. For poison analysis, we performed molecular exclusion and reverse phase chromatography, electrophoresis, and mass spectrometry. Antimicrobial, antiplasmodial, leishmanicidal, cytotoxicity, genotoxicity, and inflammatory activity of crude and/or fractions of* R. marina* secretion were also evaluated. Fractionation prior to filtration from poison showed separation of low mass content (steroids and alkaloids) and high molecular mass (protein). Material below 10 kDa two steroids, marinobufagin and desacetylcinobufagin, was detected. Crude extract and fractions were active against* Staphylococcus aureus*,* Pseudomonas aeruginosa*,* Escherichia coli*,* Plasmodium falciparum*,* Leishmania guyanensis*, and* Leishmania braziliensis*. Crude extract was also active against cancer cells although it was not cytotoxic for normal cells. This extract did not show significant DNA damage but it showed an important inflammatory effect in vivo. The information obtained in this work contributes to the understanding of the constituents of* R*.* marina *secretion as well as the bioactive potential of these molecules.

## 1. Introduction

Amphibian skin is a complex organ presenting morphological, biochemical, and physiological characteristics that exert several functions of extreme importance for the survival of these animals, such as respiration, water regulation, excretion, temperature control, reproduction, camouflage, and protection against predation and microbial/fungal infection [1].

The presence of glands in the skin is a characteristic shared by all present amphibians, constituting a synapomorphy of the class [2]. They are classified into two types of epidermal glands: mucous and granular [3]. The mucous glands, which are generally more numerous, secrete mucins, involved in lubrication and maintenance of skin moisture, thermoregulation, and osmoregulation [4, 5].

Other types, the granular or serous glands (responsible for the passive defense of the animal and generally larger than the mucous glands), have the functions of producing and storing content rich in a huge diversity of key substances for the defense system against microorganisms and predators. They are released on the animal's skin under stress conditions [6].

Historically, pharmacologic effects of amphibian secretions are described as well as their chemical compounds [1]. In particular, Anura secretions have an elevated degree of chemical complexity demonstrated by the presence of proteins, peptides, biogenic amines, and alkaloids, among other possibly unidentified classes. Several activities against bacteria, fungi, protozoa, virus, and cancer cells have been described for this type of secretion [7–9].

However, variation in the frog's diet can influence the molecules uptake by feeding, modifying the secretion composition and activity as well [10]. It leads to a continual research looking for different constituents of skin secretion in* R. marina* from distinctive places, which takes us to the objective of describing the constituents of parotid skin secretion and antiplasmodial, leishmanicidal, cytotoxicity, genotoxicity, mutagenicity, and inflammatory effects of* R. marina*.

## 2. Materials and Methods

### 2.1. Obtainment of the Crude Extract from* Rhinella marina*

The process of extraction/secretion of Bufonidae was realized by massaging and pressing the parotoids macroglandules. The collected secretion was lyophilized and stored in a freezer (-20°C) at the Animal Venom Bank (BVA) at CEBio (Center of Study of the Biomolecules Applied to Health). In order to carry out the work with the anurans, licenses were obtained from the Brazilian Institute of the Environment (IBAMA), the Chico Mendes Institute for Biodiversity Conservation (ICMBio) no. 10/2010, authorization for activities with Scientific Purpose no. 27131-1, and authorization of access to genetic heritage of the Genetic Heritage Management Council (CGEN), deliberation no. 219/2011.

For fractionation, dry and lyophilized crude venom was placed in a 100 mL flask containing 50 mL of methanol, 10 caustic soda pearls, and 1 magnetic stir bar. This solution was homogenized by cold agitation on a magnetic stirrer for about 5 minutes.

Soon after, the flask was transferred to a heating mantle; then a ball-type condenser (45 cm) was coupled to the flask. The system was maintained under reflux at 75°C for 1 hour. At that time the flask was placed on the magnetic stirrer for another 5 minutes. Then, with the aid of a pipette, only the methanol was removed, leaving the material in suspension (residual fraction).

A further 50 mL of methanol was added, and the mixture was again subjected to the magnetic stirrer for about 5 minutes and then refluxed for an additional hour. This procedure was repeated a third time.

### 2.2. Biochemical Characterization

#### 2.2.1. Filtration of Crude Extracts

Five aliquots (10 mg) of crude secretion of* R*.* marina *were solubilized in Milli-Q water, after centrifugation at 3,000g for 10 minutes, the material was filtered (AMICON < 10kDa). Two parts were obtained then: that below the cut, designated as < 10 kDa, and that retained in the filter > 10 kDa. Both materials were lyophilized and subjected to different chromatographies.

#### 2.2.2. Molecular Exclusion Chromatography

Five aliquots of 20 mg of crude extract and five aliquots of filtered above 10 kDa were solubilized in 1 mL of 20 mM Tris-HCl pH 7.4 that was shaken and centrifuged at 3,000g for 10 minutes at room temperature. They were then fractionated on Superdex Peptide 10/300 GL column (1.0 x 30 cm), previously balanced with the same buffer. The samples were eluted under isocratic gradient at flow rate of 1 mL/minute and monitored at 280 nm. The fractions were lyophilized and prepared for the next chromatographic step.

#### 2.2.3. Reverse Phase Chromatography

Fractions and filtrate under 10 kDa were resuspended in 1 mL of 0.1% Trifluoroacetic Acid (TFA) under shaking and thereafter centrifuged for 10 minutes at 3,000g. After centrifugation, the supernatant was chromatographed on Phenomenex Jupiter Analytical C-18 reverse phase HPLC (250 x 10.00 mm, 5 *μ*m), balanced with 0.1% (v/v) TFA and eluted with 99.9% acetonitrile/0.1% TFA solution. At a flow rate of 1 mL/minute, fractions were collected manually and monitored at 215 nm and 280 nm.

According to the yield of each extract and fraction, the tests and concentrations were chosen. Not all compounds were tested in all bioassays.

#### 2.2.4. Protein Quantification

The method used for protein quantification was that of Lowry et al. [11], with modifications. The reagents used were Bio-Rad's DC Protein Assay Kit where 10 *μ*L of fractions of* R*.* marina *in triplicate, 100 *μ*L of reagent A, and 800 *μ*L of reagent B were incubated for 15 minutes. The reading was monitored at 750 nm by BioMate 3 spectrophotometer. The standard curve was previously prepared using bovine albumin (BSA) and stock solution of 2 mg/mL, diluted in several concentrations ranging from 0.125 to 1.5 mg/mL. The reading of the dilutions resulted in a linear correlation coefficient of 0.999 for the standard curve.

#### 2.2.5. One-Dimensional SDS-Polyacrylamide Gel Electrophoresis (1D SDS-PAGE)

SDS-PAGE was performed as described by Laemmli [12], with modifications. The fraction from affinity chromatography was applied on a discontinuous gel having dimensions of 180 x 160 x 1.0 mm. The sample was preheated in a water bath at 95°C for 5 minutes. The system was formed by a 3.5% (w/w) acrylamide concentrator gel, 0.5 M Tris-HCl buffer (pH = 6.8), 12.5% (m/v) acrylamide buffer gel, and 1.5 M Tris-HCl buffer (pH = 8.8). The run buffer solution used to fill the well reservoirs was composed of 0.06 M Tris-Base, 0.5 M glycine, and 0.15% (m/v) SDS. In the electrophoretic run, the current of 25 mA per gel and power was fixed 5 W until the entire gel was traversed.

At the end of the electrophoresis the gel was washed with Milli-Q water for 5 minutes to remove excess SDS. Thereafter, it was subjected to the fixative solution for 1 hour and then stained for 2 hours under gentle shaking using Coomassie G-250 at 1% (w/v), prepared with 45% (v/v) of methanol, 45% (v/v) of deionized water, and 10% of glacial acetic acid.

#### 2.2.6. Mass Spectrometry

The experiments were carried out in the Laboratory of Biochemistry and Biophysics of the Butantan Institute. The apparatus used was ESI-IT-TOF mass spectrometer (Electrospray-Ion Trap-Time of Flight) (Shimadzu Co., Japan). The chromatographic fractions were diluted in 50 *μ*L of 0.1% acetic acid and automatically introduced into the mass spectrometer, with the spray voltage at 4.5 KV and capillary voltage 1.76 KV at a temperature of 200°C. The spectra were obtained in positive mode in the range of 50-2000 m/z. The fragmentation was obtained by argon collision gas, with 50% of energy. LabSolutions software (LCMS solution 3.60.361, Shimadzu) was used to process the data. The generated MS/MS spectra were analyzed by Peaks Mass Spectrometry software (Bioinformatics Solutions Inc., Canada).

### 2.3. Biological Activities

#### 2.3.1. Antimicrobial Assay

Microbiological analysis was defined by the Minimum Inhibitory Concentration (MIC), which consists of the lowest concentration of the poison or molecule capable of inhibiting bacterial proliferation. MIC was determined by the microdilution susceptibility test described by Cockerill et al. [13] with adaptations. The bacterial strains used were provided by the Gram-negative bacteria* Escherichia coli* (ATCC 25922) and* Pseudomonas aeruginosa* cultures (ATCC 27853) by the Research Center for Tropical Medicine of Rondônia (CEPEM). Gram-positive* Staphylococcus aureus* (ATCC 29213) was cultured in Luria Bertani broth (LB) for 24 hours (exponential phase) and adjusted to a turbidity absorbance corresponding to 0.5 of the McFarland scale, corresponding to 1.5 x 106 colony forming units (CFU)/mL. The crude poison and chromatographic fractions of* R. marina* were applied to each well of the microplate (96 wells): 50 *μ*L of sample and 150 *μ*L of bacterial suspension (1.5 x 106 CFU/mL) of* S. aureus*,* E. coli*, and* P. aeruginosa* separately, totaling 200 *μ*L for each well, in four concentrations, ranging from 3.12 to 20 *μ*g/mL. The experiment was carried out with LB broth, the negative control was done with the bacterial suspension in LB broth, and the positive control with Chloramphenicol (20 *μ*g/mL) was added to the bacteria in culture medium. Bacterial growth was determined by spectrophotometry at the wavelength of 630 nm (TC 96 Elisa Microplate Reader).

#### 2.3.2. Antiplasmodial Assay

The culture of Chloroquine-resistant strain (W2) of* P. falciparum *was maintained in the laboratory conditions as described by Trager and Jensen [14] at 5% hematocrit (human type O-positive red blood cells) in complete RPMI 1640 medium (supplemented with 25 mM HEPES, 21 mM sodium bicarbonate, 11 mM glucose, 40 *μ*g/mL gentamicin, and 10% (w/v) Albumax). The culture flasks were maintained in gas mixture of 5% O2, 5% CO2, and 90% N2. The culture, with predominance of ring stages, was utilized for chemotherapy assays. It was obtained by synchronization with sorbitol, as described by Lambros and Vanderberg [15]. After synchronization, 180 *μ*L/well of the culture containing 0.5% parasitemia and 1.5% hematocrit was distributed in 96-well microplates. Previously, 20 *μ*L of the crude and methanol extracts were added in triplicate, on serial concentrations (1.56 to 50 *μ*g/mL) as well as the positive control, artemisinin (1.56 to 50 ng/mL). The fluorescence assay was performed as recommended by Smilkstein et al. [16]. Briefly, after 48 hours of incubation in desiccators, the supernatant of the test plates was discarded and washed with 1x PBS (137 mM NaCl, 10 mM phosphate, 2.7 mM KCl at pH 7.4). Subsequently, the red blood cells sedimentation was diluted in 100 *μ*L of SYBR Green I-containing lysis buffer. Immediately after lysis of the red blood cells, 100 *μ*L of the cells lysate was transferred to a flat bottom plat containing 100 *μ*L 1x PBS. These plates were incubated for 30 minutes at room temperature, protected from light. Fluorescence data were acquired using microplate reader spectrophotometer (Synergy, Biotek) at 590 nm emission and 485 nm excitation.

#### 2.3.3. Leishmanicidal Assay

Strains of* Leishmania guyanensis *(IOCL 565) and* Leishmania braziliensis *(IOCL 566) were provided by Leishmania Collection from the Oswaldo Cruz Institute (CLIOC-RJ);* Leishmania spp*. promastigote forms were cultured at 24°C in RPMI medium, supplemented with 20% FBS, 2 mM L-glutamine, 20 mM HEPES, and 40 *μ*g/mL of gentamicin. The promastigote cultures were maintained in vitro using a parasitic aliquot in stationary growth phase, which was diluted in erythrosin B dye (0.04%). The concentration of the protozoa was estimated with the aid of a Neubauer chamber and adjusted to 1 x 106 promastigotes per mL. The parasites that were stained red were considered dead and those that were birefringent and mobile were considered alive. Only the living parasites were quantified. Consequently, the parasites were placed in RPMI/FBS culture medium, maintained at 24°C, and subcultured every five days for up to eight passages. The cytotoxicity of crude and methanol extracts was determined using the viability of promastigote forms of* L. guyanensis* and* L. braziliensis* with an MTT assay [17]. For these experiments, promastigotes in the stationary phase were seeded at 5 x 105 parasite/100 *μ*L/well on a 96-well plate in complete RPMI 1640 medium with serial dilutions of the extracts (1.56 to 100 *μ*g/mL), and Pentamidine (3 *μ*g/mL) was used as positive control. Promastigotes were incubated at 23°C for 72 hours. Then, 10 *μ*L of MTT solution (50 mg/mL 1x PBS) was added to each well, incubated for more than 4 hours at 23°C, and then centrifuged at 8000 x g for 10 minutes. In order to dissolve the formazan crystals formed, each well received 100 *μ*L of DMSO and was incubated for 1 hour at room temperature. The absorbance was immediately read at 540 nm in spectrophotometer. Each concentration was screened in triplicate and experiments were repeated three times.

#### 2.3.4. In Vitro Antitumoral Assays

The human cell lines MRC5 (lung normal fibroblast), HepG2 (hepatoma), HCT116 cell (colorectal carcinoma), and HT29 cell (colorectal adenocarcinoma) were used. The cells were acquired from American Tissue cell culture (HCT116 and HT29), Rio de Janeiro Cell Bank (HepG2), or kindly provided by Dr. Alain Sarasain (Institut de Cancérologie Gustave Roussy, CNRS, Villejuif, France). All cell lines were grown in DMEM supplemented with 10% FBS, 100 units/mL−1 penicillin and 100 *μ*g/mL−1 streptomycin at 37°C in a humidified atmosphere of 5% CO2. Cells (5 x 105 cells) were seeded in complete media and grown for one day prior to treatment with crude poison using different concentrations (12.5 to 100 *μ*g/mL) before evaluation by MTT and comet assay. The cells were treated for 72 hours under standard conditions.

#### 2.3.5. MTT Assay

MTT reduction was performed according to Denizot and Lang [18]. Briefly, after treatments, cells were washed once with PBS before the addition of 0.1 mL serum-free media containing yellow tetrazolium salt (MTT; 1 mg/mL) dye and incubated for 3 hours at 37°C. After incubation, the supernatant was removed, the residual purple formazan product solubilized in 200 *μ*L DMSO and stirred for 15 minutes, and its absorbance measured at 570 nm (Spectra Max M2e, Molecular Devices). The absorbance of negative control cells was set as 100% viability, and the values of treated cells were calculated as percentage of control.

#### 2.3.6. Comet Assay

The alkaline version of the comet assay, which was performed according to the method of Singh et al. [19] with minor modifications according to Speit and Hartman [20], was used to detect DNA damage after crude extract treatment. Briefly, an aliquot (10 *μ*L) of cell suspension was mixed with 0.75% low-melting-point agarose and cast on to microscope slides precoated with 1.5% normal agarose. Slides were incubated in ice-cold lysis solution. After lysis for 72 hours, the DNA was allowed to unwind for 15 minutes in electrophoresis solution (300 mM NaOH, 1 mM EDTA, pH~13). Electrophoresis was conducted at 4°C for 15 minutes at 0.94 V/cm. The slides were then neutralized with tris buffer (0.4 M Tris, pH 7.5) and stained with silver nitrate. For DNA damage evaluation, 100 cells per sample were analyzed by optical microscopy. Cells were visually scored by measuring the DNA migration length, and the amount of DNA in the tail was divided into five classes, from undamaged (0) to maximally damaged (4), and a damage index (DI) value was calculated for each sample. Damage index therefore ranged from 0 (completely undamaged: 100 cells × 0) to 400 (with maximum damage: 100 cells x 4). The MMS (40 *μ*M) was used as the positive control.

#### 2.3.7. Mutagenesis in Saccharomyces cerevisiae

The* S. cerevisiae* strain XV185-14c (MAT ade2-2 arg4-17 his1-7 lys1-1 trp5-48 hom3-10) was used in the mutagenicity assay. Briefly, a suspension of 2 x 108 cells/mL in exponential growth phase was incubated in PBS for mutagenic evaluation with different concentrations of* R. marina* poison at 30°C for 18 hours. Cell survival was determined in SC (3–5 days, 30°C), and mutation induction (LYS, HIS or HOM revertants) in appropriate omission media (5–7 days, 30°C). Whereas his1–7 is a nonsuppressible missense allele and reversions result from mutations at the locus itself, lys1-1 is a suppressible ochre nonsense mutant allele, which can be reverted either by locus-specific or by forward mutation in a suppressor gene [21]. True reversions and forward (suppressor) mutations at lys1-1 locus were differentiated according to Schuller and von Borstel [22]. Assays were repeated at least three times, and plating was performed in triplicate for each dose.

#### 2.3.8. In Vivo Assays

*Animals*. Male Swiss mice (18–20 g) were used. These animals were housed in temperature-controlled rooms and received water and food ad libitum until used. These studies were approved by the Experimental Animals Committee of IPEPATRO in accordance with the procedures laid down by the Universities Federation for Animal Welfare.

*Induction of Inflammatory Reaction*.* Rhinella marina *crude extract (20 *μ*g/mL), dissolved in 1 mL sterile phosphate-buffered saline (PBS), pH 7.2, was injected by the intraperitoneal (i.p.) route. Control animals received 1 mL of sterile PBS. After 6 hours of these injections, the animals were killed under halothane atmosphere and the inflammatory exudate was withdrawn after washing the cavities with 3 mL phosphate-buffered saline (PBS), pH 7.2 containing heparin (10U/mL). Aliquots of the washes were used to determine total cell counts.

*Leukocyte Harvesting and Counting*. Leukocytes were harvested by washing peritoneal cavities with 3 mL of PBS containing heparin (10 U/mL). Aliquots of the washes were used to determine total cell counts in a Neubauer chamber after dilution (1:20, v/v) in Turk solution (0.2% crystal violet dye in 30% acetic acid). For differential cell counts, cytospin preparations (400 xg/5 min) were stained with Panoptic stain. Differential cell counts were performed by counting at least 100 cells, which were classified as either polymorphonuclear or mononuclear cells, based on conventional morphological criteria according to Zuliani et al. [23].

*Phagocytic Activity*. In brief, leukocytes from animals that received* R*.* marina *crude extract or PBS (control animals) according to item 5.4.2 were harvested according to item 5.4.3, adjusted to 2x105/200 mL, and incubated with nonopsonized zymosan particles (2x106) diluted in RPMI culture medium supplemented with gentamicin (100 *μ*g/mL), L-glutamine (2 mM), and 10% of fetal bovine serum for 40 minutes at 37°C and 5% CO2 atmosphere. After that, an aliquot of 50 *μ*L was used to prepare glass slides after cytospin preparation (400 xg/5 minutes) and stained with Panoptic. The extent of phagocytosis was quantified by contrast phase microscopic observation. At least 100 macrophages were counted in each determination and those containing three or more internalized particles were considered positive for phagocytosis. Results were presented as the percentage of cells positive for phagocytosis.

*Superoxide Anion Production*. A suspension of leukocytes from animals that received* R*.* marina *crude extract or PBS (control animals) according to item 5.4.2 was collected, according to item 5.4.3, adjusted to 2x105/200 *μ*L, and incubated for 1 hour with 0.1% nitroblue tetrazolium (NBT) at 37°C and 5% CO2 atmosphere. After incubation, the leukocytes were centrifugated (400 xg/5 min) in cytospin. The slides were fixed with 100% methanol for 5 minutes and stained with 1% safranin for 5 minutes. After that, the slides were washed with distilled water. The extent of superoxide was quantified by microscopic observation. At least 100 leukocytes were counted in each determination and those containing crystals of formazan were considered positive for superoxide production. Results were expressed as percentage of cells positive for superoxide anion production.

## 3. Results

### 3.1. Biochemical Profile of Rhinella marina Poison (<10 kDa)

**Figure 1:**
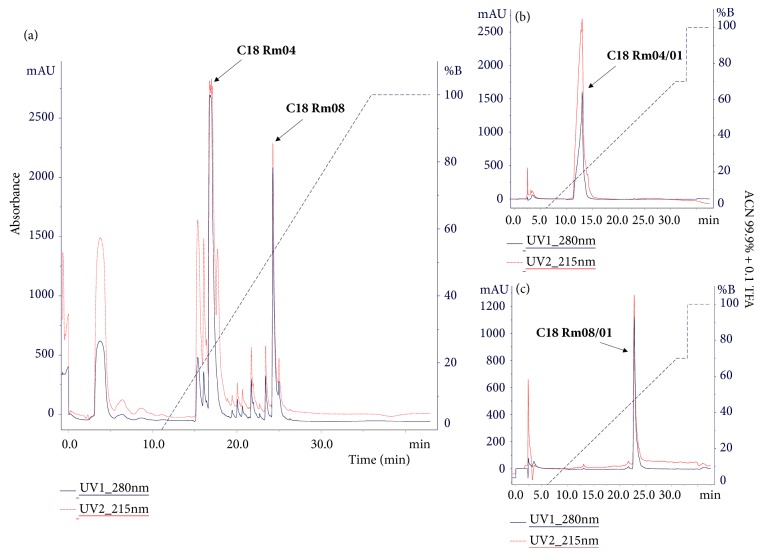
(a) Chromatographic profile of the filtrate (below 10 kDa) on reverse phase C18 column. The separation of the peaks C18 Rm-04 and Rm-08 ((b) and (c)) is observed. Linear gradient of 99.9 + 0.1% AC/TFA, monitoring at 280 and 215 nm absorbance.

The filtered material (<10 kDa) submitted to reverse phase chromatography (Figure 1(a)) showed 6 main peaks. Two were rechromatographed (Figures 1(b) and 1(c)), apparently with possible isolation of the compounds in the fractions.

**Figure 2:**
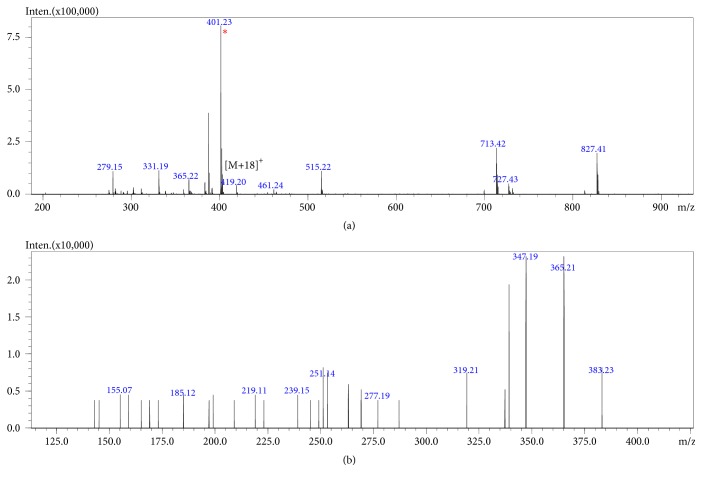
Mass spectrometry profile (ESI-IT-TOF) of fraction Rm-04 (<10 kDa). Profile compatible with molecular weight of the steroid desacetylcinobufagin mass band of 400 Da (a), with protonated precursor 401 and fragments 383, 365, 319, and 251 [M + H]+(b).

**Figure 3:**
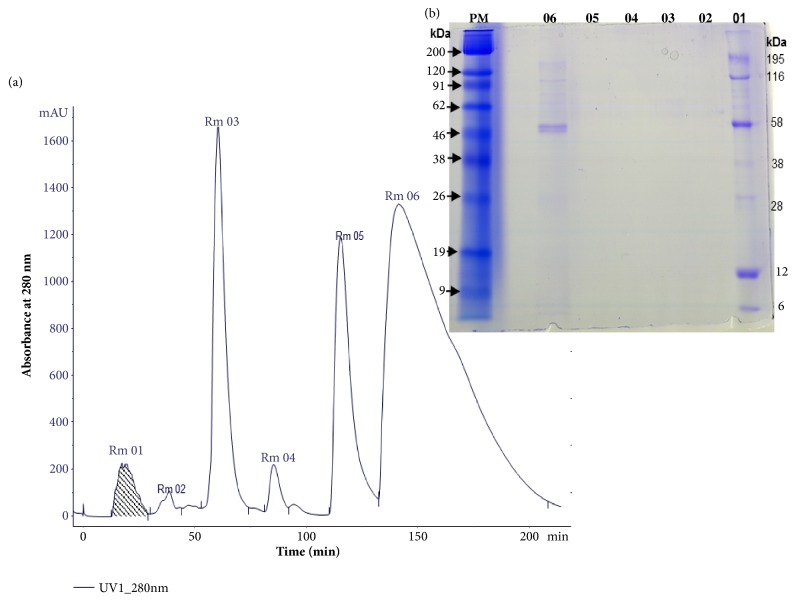
Chromatographic profile of crude* Rhinella marina* poison and electrophoretic profile of fractions from molecular exclusion chromatography. (a) Chromatographic profile of the crude poison of* R. marina* in a column (Superdex Peptide 10x30 cm) of molecular exclusion. The interest fraction Superdex Rm01 and its electrophoretic profile in reducing conditions. (b) MW: 200 kDa (Myosin); 120 kDa (*β*-Galactosidase); 91 kDa (Bovine Serum); 62 kDa (Glutamate); 46 kDa (Ovalbumin); 38 kDa (Carbonic anhydrase); 26 kDa (Myoglobin); 19 kDa (lysozyme); and 9 kDa (Aprotinin).

The fractions (C18Rm04 and C18Rm08) analyzed by mass spectrometry (ESI-IT-TOF) revealed a molecular mass corresponding to steroids, as demonstrated by the spectrum of Figures 2 and 3.

The fragmentation profile of the 401.23 m/z ion (Figure 1(b)) shows fragments with successive losses of H_2_O (18 Da), CO (28 Da), and possible elimination of *α*-purine (96 Da), according to Table 1, characterizing the bufadienolide desacetylcinobufagin [11].

The fragmentation profile (Figure 2(b)) demonstrated compatible ions with the steroid marinobufagin (bufadienolide), with protonated precursor 401.23 m/z. Successive losses of H_2_O and CO (Table 2) generate fragments 383, 365, 347, 319, and 291 m/z, respectively [11–13].

### 3.2. Biochemical Profile of Rhinella marina Poison (>10 kDa)

The crude extract submitted to exclusion chromatography presented 6 main fractions (Figure 3(a)), named Superdex Rm-01 to Rm-06, respectively, at the elution time.

The electrophoresis of the fractions under reducing conditions revealed a higher concentration of proteins in the fraction Superdex Rm-01, where it is possible to evidence the presence of protein bands in the apparent mass range of 6 to 195 kDa and a visible band in the fraction Superdex Rm-06 with apparent mass of 48 kDa, while other fractions (Rm-02/Rm-05) showed no visible protein bands. They are probably low mass molecules such as alkaloids and steroids (Figure 3(b)).

The profile showed similarity with three molecular masses described in the biochemical characterization of* R. marina* secretion (Table 3).

**Figure 4:**
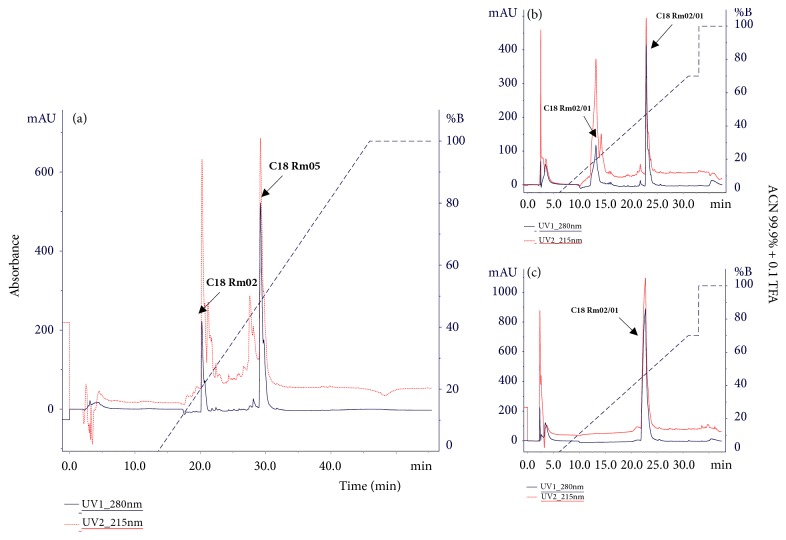
(a) Chromatographic profile of the fraction Rm 01 (Gel Filtration) on reverse phase C18 column. The separation of the peaks C18 Rm02 and Rm 05 is highlighted by the arrows. (b) Rechromatography of the fraction C18 Rm01/02 and C18 Rm01/05. (c) 99.99 + linear gradient 0.1% ACN/TFA, 280 nm absorbance monitoring for the detection of aromatic amino acid side chains (Phenylalanine, Tyrosine, and Tryptophan). The purification profile of the sample is highlighted.

The second step of the fractionation was performed in reverse phase liquid chromatography (RP-HPLC) of the Rm-01 fraction. In this step, two main fractions denominated C18 Rm-02 and C18 Rm-05 were obtained (Figure 4(a)).

The third step determined the reliability of purity. The C18 Rm-02 fraction presented two new fractions (Figure 4(b)) and the C18 Rm-05 fraction presented a single peak (Figure 4(c)).

### 3.3. Biological Activity

In the antimicrobial assay, the crude extract showed activity against* S. aureus* and* P. aeruginosa*. The fraction Rm-01 and DCCB exhibited MIC < 3.12 *μ*g/mL against all the microorganisms tested. Marinobufagin inhibited* S. aureus* although it hast not been tested against other bacteria (Table 4).* E. coli* was the most sensitive bacterium to fractions Rm.

The crude extract of* R. marina* showed the lowest IC_50_ against* P. falciparum* (2.43 *μ*g/mL), while the methanolic extract presented the lowest IC_50_ against* L. guyanensis* (3.99 *μ*g/mL) (Table 5).

**Figure 5:**
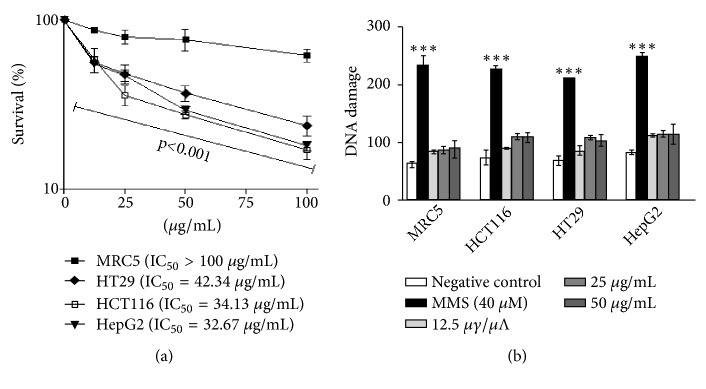
(a) Cytotoxic effects of crude venom upon human cells, after 72 h treatment, by MTT assay. Results are expressed as mean percentage in treated cells compared to control (solvent) ± SD of three independent experiments. In detail the IC50 value for each cell line. (b) Induction of DNA strand breaks by crude venom as evaluated by the comet assay in alkaline conditions. Bars represent the mean ± SD of three independent experiments. MMS is used as positive control. ^∗^Significant difference as compared to negative control treatment at ^∗∗∗^P < 0.001/one-way ANOVA Tukey's multiple comparison test.

Figure 5(a) shows the cytotoxic effects of* R. marina* crude poison on human cell lines. The poison induced concentration-dependent cytotoxic effect in all tested tumoral cell lines after 72 hours of treatment, while for the nontumoral MRC5 a small reduction in viability was observed, indicating a selective cytotoxic effect for tumor cells. Interestingly, there was no significant increase in the induction of DNA damage, which indicates an absence of genotoxicity of the poison (Figure 5(b)).

In the same way, the poison did not induce locus-nonspecific, locus-specific, or frameshift mutations in XV145-14c* S. cerevisiae* strain in any of the concentrations employed (Table 6)

**Figure 6:**
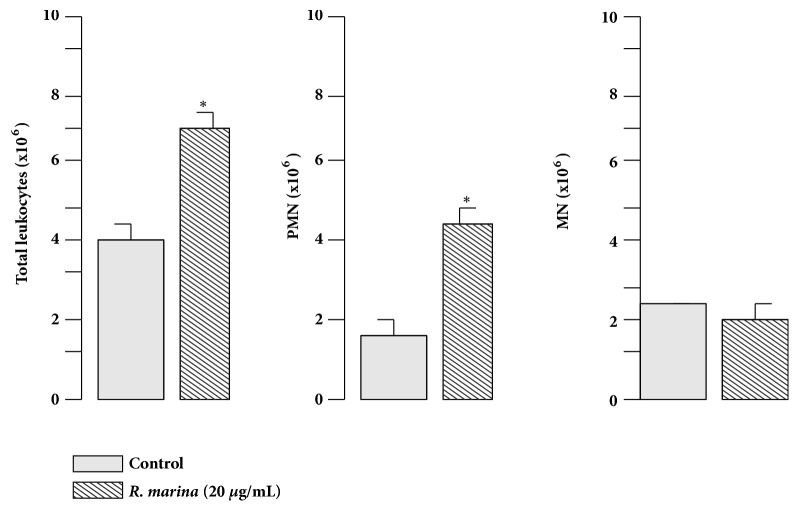
Effect of* Rhinella marina* poison on inflammatory reaction.

*Rhinella marina* crude extract induced a pronounced cellular influx into mice peritoneal cavity (Figure 6). Differential cell counts showed that the predominant cells in the infiltrate after* Rhinella marina* crude extract injection were polymorphonuclear leukocytes (PMN), mainly neutrophils, whereas the population of mononuclear leukocytes (MN) did not show a significant increment (Figure 6). Since PMN influx was observed 6 hours after* Rhinella marina* crude extract intraperitoneal injection, we evaluated the phagocytosis and production of superoxide production at this time point.

**Figure 7:**
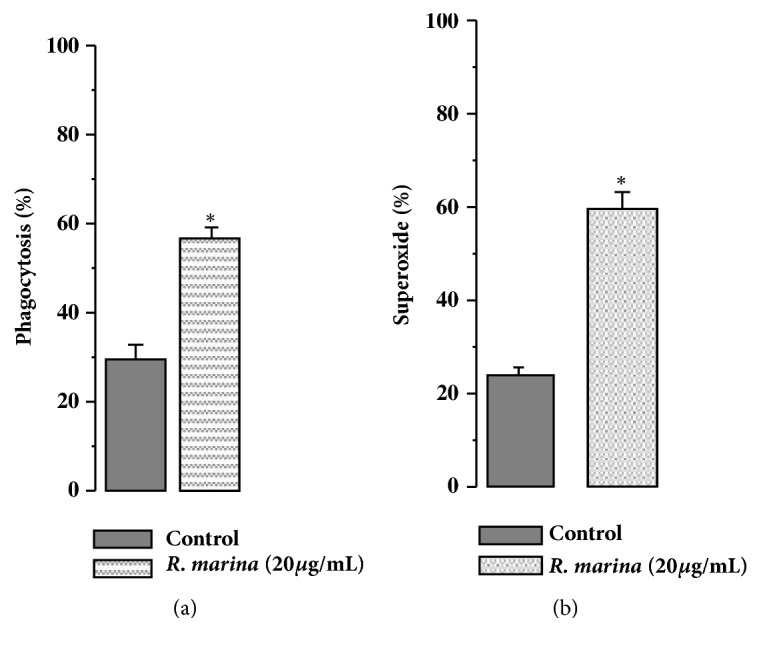
(a) Effect of* Rhinella marina* poison on leukocytes phagocytosis. (b) Effect of* Rhinella marina* poison on superoxide anion.

As shown in Figure 7, control peritoneal leukocytes showed a percentage of phagocytosis (Figure 7(a)) and superoxide anion production (Figure 7(b)) of about 25 and 22%, respectively. After* Rhinella marina* crude extract injection, about 58% of harvested peritoneal leukocytes phagocytosed zymosan particles and 60% of the same type os cells produced superoxide, which was statistically different from controls.

## 4. Discussion

The secretion of the skin and/or amphibian glands is rich in bioactive substances with great potential for the development of new pharmacological tools, an important source of biologically active chemical compounds against bacteria, fungi, protozoa, viruses, and cancer cells [7–9]. However, for possible applications only with the characterization this can be evidenced.

Poison purification strategy has taken into account the separation of proteins from the low molecular mass compounds by filtration steps with 10 kDa cut that has been used in the literature, for example, to isolate serine protease inhibitors from cutaneous secretion of the* Bombina microdeladigitora* anuran [24] and to separate peptides and steroids from the parietal secretion of* R*.* marina* [25].

Based on the studies of Ye and Guo [26] molecular weight compatible with cardiotonic steroids, classified as bufadienolides, commonly found in Bufonidae anurans [4], was obtained from fragmentation profile analysis (Figures 2(b) and 3(b)).

The steroids marinobufagin and desacetylcinobufagin, identified in this study, although possessing similar molecular mass, differ in R2 and radicals, alternated by a hydroxyl group [26].

In this study, there was no molecular weight detection similar to or compatible with peptides. However, Rash et al. [25], through mass spectrometry analysis, demonstrated a low amount of peptides in the parotoids secretion of* R*.* marina* (specimens introduced in Australia).

Species of the genus* Rhinella* have raised great interest in the compounds of the skin and macroglands. Riera et al. [27] isolated two 50 kDa ligand (LBP1) ligand proteins (56 Lbs) from* R*.* arenarum* skin. More recently, various proteins have been characterized from the skin extract of the Asian species,* Bufo andrewsi*, such as the trypsin inhibitor with relative mass of 22 kDa; irreversible serine protease inhibitor (60 kDa), anti-HIV protein (63 kDa), and lysozyme with 15 kDa [28–32]. In another Asian species, a high mass protein (79 kDa) called BMP1 was isolated from* Bufo melanostictus* [32].

The action of the antimicrobial activity in this work was demonstrated according to observations in other studies with skin secretions and/or macroglands of anurans. In fact, it is characteristic to find molecules that present such activity contained in the skin, poison glands, and stomach tissue of some anurans, especially of the genera* Xenopus*,* Bombina*,* Phyllomedusa*,* Lithobates*, and* Rhinella* [33, 34].

As a result of anurans secretion of the* Rhinella* group, several biological activities are verified due to the steroidal compounds, together with the alkaloids studied in Bufonidae, with respect to biochemical, pharmacological, and biological characterization. This may be related to the fact that the most abundant components in the secretion on steroids are the bufadienolides (cardiotonic steroids) that present several biological and pharmacological activities, for example, antiviral [35], and the use of the antigen-binding enzyme (GABA) and other insecticides [36].

Regarding the secretion of* R*.* marina* observed crude extract, chromatographic fractions and isolated compounds attributed to the gradual interference in microbial growth. In this process, the bacteriostatic action of antimicrobial agents tested was verified. Chloramphenicol was used as control. It acts in the inhibition of the bacterial protein synthesis, interfering in the growth or replication [37].

In this study, antimicrobial activity in strains of* S*.* aureus*,* P*.* aeruginosa*, and* E*.* coli*, with lower concentrations and incubation time was found in the study for skin extracts and with secretion of the macroglandules of* Rhinella icterica*, with MIC between 3.125 and 25 *μ*g/mL, capable of inhibiting the colony forming unit (CFU) by at least 50% in a 24-hour incubation. Pinto et al. [38] demonstrated antimicrobial action on* S*.* aureus* (strains ATCC 25923) and* E*.* coli* (ATCC 25922), being concentration dependent (doses greater than 50 mg/mL) with time of exposure to the poison from 15 to 30 minutes.

In the poison of* R*.* marina*, two steroids demonstrated antimicrobial activity: marinobufagin [39, 40] and desacetylcinobufagin [26]; isolates of* R*.* rubescens* showed total inhibition of growth at the concentration of 128 *μ*g/mL for* S*.* aureus* growth (ATCC 25213) and 16 *μ*g/mL for* E*.* coli* (ATCC 25922). However,* Bufo* species have other molecules capable of inhibiting bacterial growth, such as bufotenin, an alkaloid that acts on Gram-positive and Gram-negative bacteria, and some fungi [7]. In addition, *β*-galactose-binding lectins, proteins, were described and isolated from the skin extract of the species* R*.* arenarum*, inhibiting the growth of* E*.* coli*,* Enterococcus faecalis*, and* Proteus morganii* [27].

In relation to mechanism of action, isolated marinobufagin acts as a potent inhibitor of the Na+ K+ ATPase pump and causes substantial vasoconstriction in human and mouse blood vessels [41]. However, no such activity attributing to the steroid desacetylcinobufagin was found in the literature.

Studies with the secretion of* R*.* marina* have been shown to be promising, as it has been reported in the literature, with lethal effects on HL-60 leukemia tumor cells [40]. Several bufadienolides show antitumor activity: in colon (26-L5, CT26.WT), leukemia (K562, U937, ML1), and melanoma (MDA/MB-435, B16/F10, SKMEL-(PLC/PRF/5)) and in the absence of other factors such as the presence or absence of the disease.

Biological activity against* P*.* falciparum* of the crude extract was considered particularly high (IC_50_ = 2.14 *μ*g/mL) in relation to methanol extract. It corroborates with a previous study of the crude extract against W2 strain (IC_50_ = 0.53 *μ*g/mL) of* R*.* marina* poison [42]. The high activity of toad secretions is related to the presence of antimicrobial peptides in its composition [43]. Buforin II is a peptide isolated from* Bufo gargarizans* stomach and displays a well-known antimicrobial and antiplasmodial activity in Bufonidae family [44, 45]. Dermaseptin-type peptides, isolated from* Phyllomedusa* sp. skin, have also shown potent and fast antiplasmodial effect in vitro, resulting in derivations searching for more effective and less hemolytic and cytotoxic molecules [46].

Biological activity against Leishmania of the crude poison of* R*.* marina* showed growth inhibition against* L*.* braziliensis* (14.82 *μ*g/mL) and* L*.* guayanensis* (9.34 *μ*g/mL) promastigotes, when compared to ethanolic extract, showing inhibition of* L*.* guayanensis* (3.99 *μ*g/mL), but not showing effectiveness against* L*.* braziliensis* at the concentration of 100 *μ*g/mL.

Tempone et al. [47] reported antiparasitic activity of the genus* Rhinella*. This study describes the action of steroids isolated from the crude extract of* R*.* jimi* (telocinobufagin and hellebrigenin) with leishmanicidal activity against* L*.* infantum* promastigotes with IC_50_ of 126.2 and 61.2 *μ*g/mL. Only the hellebrigenin steroid was active against and trypomastigote of* T*.* cruzi* with IC_50_ of 91.75 *μ*g/mL. The authors suggest that the activity of these steroids may involve mitochondrial degradation and perturbation of the parasite membrane, resulting in cell death.

In this context, Schmeda-Hirschmann et al. [48] describe 29 compounds isolated and identified from the crude poison of* R*.* marina*. In this study, poison showed antiproliferative effect against pulmonary fibroblasts and human breast cancer cells mediated by the production of ROS.

The use of secretions from the parotid glands of Bufonidae is common in Asian and American tribes in folk medicine. Chemical constituents existing in the Bufonidae family present therapeutic potential for the treatment of allergies, inflammations, cancer, infections, and other diseases. This report highlights the importance of Bufonidae species such as* R. marina* in the prospection of chemical components, bioactive and microbial peptides effective in the search for new drugs and therapies [40, 49].

The cytotoxicity of the* R*.* marina* poison was observed in tumoral cell lines but not in nontumoral cell line. This effect cannot to be associated with an effect on the DNA molecule; since there is an increase in cytotoxic effects, the incidence of DNA damage evaluated by the comet assay does not occur.

Some studies have shown that components of other species of* Rhinella* have an antitumor effect. According to Sakate and Oliveira [50], the substances that make up the frog poison can be divided into basic compounds (biogenic amines) and steroid derivatives. Hellebrigenin and telocinobufagin are basic compounds that have been isolated from skin secretions of* Rhinella rubescens*, which have shown potent antitumor activity against liver carcinoma.

Buforin, a cardiotonic steroid found in frogs of the Bufonidae family, has induced apoptosis in leukemia cells and has inhibitory properties of malignant melanoma cells and squamous epithelial cell activity [47]. Cunha-Filho et al. [36] studied the effect of* Rhinella schneideri* poison on melanoma, leukemia, glioblastoma, and colon tumor cell lines. It was observed that four compounds isolated from this poison (bufalin, telocinobufagin, hellebrigenin, and *β*-sitosterol) present potent inhibitory activity against these cell lines. *β*-sitosterol compound showed the highest antiproliferative activity in all strains.

In another study on the poison of* Bufo marinus* (*R*.* marina*), it was shown that the peptides present in very low concentrations, which may be compounds derived from the degradation of proteins involved in the cellular maintenance of the parotid gland, may contribute to the toxicity caused by poison toad [25].

Interestingly, in the genotoxicity and mutagenesis assays, no significant DNA damage induction results were found, which, despite not explaining the cytotoxicity of the compound in the tumor cells, brings interesting data on the safety of* R*.* marina* poison use considering its numerous pharmacological functions, some of which are presented here.

In this study, the inflammatory effects induced by* R*.* marina* crude extract in mice animal model were investigated. Results showed that, 6h after intraperitoneal injection of* R*.* marina* crude extract, an important cellular influx was observed, mainly composed by polymorphonuclear cells. It is interesting that* R*.* marina* crude extract has also the ability to activate these cells, polymorphonuclear, and to phagocytose and produce superoxide anion production. There is no data in the literature showing similar results which is the first demonstration.

## 5. Conclusions

*R*.* marina* poison proved to have activity against a number of organisms. We can highlight, in particular, the first descriptions of the activity of crude poison, DCCB, and marinobufagin isolates against bacteria. In addition, the leishmanicidal activity, cytotoxic effect against cancer cells, and induction of the inflammatory response in the murine model caused by the crude poison of* R*.* marina* are highlighted. These results amplify the range of bioactive molecules and possible applications for* R. marina* poison and components.

## Figures and Tables

**Table 1:** Analysis of the ion fragmentation pattern 401.23 m/z (Figure 1(a)).

**[M+18]** ^**+**^	**[M+H]** ^**+**^	**a**	**b**	**c**	**d**	**e**

419	401	383	365	347	319	251

**[M+H]** ^**+**^ **-a**	**a-b**	**b-c**	**c-d**	**c-e**	**Identification**	

18	18	18	28	96	Desacetylcinobufagin	

**Table 2:** Analysis of the ion fragmentation pattern 401.23 m/z (Figure 2(a)).

**[M+18]** ^**+**^	**[M+H]** ^**+**^	**A**	**b**	**c**	**d**	**e**

419	401	383	365	347	319	291

**[M+H]** ^**+**^ **-a**	**a-b**	**b-c**	**c-d**	**d-e**	**Identification**	

18	18	18	28	28	Marinobufagin	

**Table 3:** Comparison of apparent molecular weight on SDS-PAGE.

**Apparent molecular weight (kDa)**		
**Present study** ^**1**^	[14]	[15]

195	-* *-	-* *-
116	116	116
58	55	55
38	-* *-	-* *-
30	30	30
12	-* *-	-* *-
6	-* *-	-* *-

^1^The fraction from the chromatography of molecular exclusion (Rm01) compared with molecular mass described by other authors of the poison of Rhinella marina.

**Table 4:** Antimicrobial activity of crude and isolated compounds of *Rhinella marina*.

Microorganisms	Crude Extract (*μ*g/mL)	Fractions Rm (*μ*g/mL)						DCCB (*μ*g/mL)	Marino (*μ*g/mL)
		01	02	03	04	05	06		
*S. aureus*	21	*∗*	*∗*	5,65	19,7	>25	>25	*∗*	*∗*
*P. aeruginosa*	10,79	*∗*	>25	>25	>25	>25	>25	*∗*	NT
*E. coli*	>25	*∗*	*∗*	*∗*	*∗*	*∗*	*∗*	*∗*	NT

Note. *∗*: corresponding MIC <3,12. NT: no test.

**Table 5:** Antiparasitic activity and cytotoxicity of crude and methanolic extract of *Rhinella marina*.

Extracts	Anti-parasitic activity IC_50_ (*μ*g/mL)		
	*P. falciparum*	*L. braziliensis*	*L. guyanensis*

Crude	2.43 ± 1.2	14.82 ± 2.42	9.34 ± 4.50
Methanolic	12.04 ± 1.27	≥ 100	3.99 ± 1.23

**Table 6:** *Rhinella marina* crude extract induced reversion of point mutation for his1–7, frameshift mutation (hom3–10), and ochre allele (lys1–1) in haploid strain XV185-14c of *Saccharomyces cerevisiae* in stationary phase.

	Treatment	Survival (%)	*LYS1*/10^7^survivors^b^	*HIS1*/10^7^survivors^a^	*HOM3*/10^7^survivors^a^

Negative control	0	100.00	0.87 ± 0.69^c^	0.62 ± 0.35^c^	0.11 ± 0.08^c^
4-NQO^e^	0.5 *μ*g/mL	15.33^∗∗∗^	125.92 ± 9.19^∗∗∗^	151.88 ± 6.3^∗∗∗^	41.22 ± 8.50^∗∗∗^
*Rhinella marina* crude poison	10 *μ*g/mL	97.04	0.45 ± 0.32	0.627 ± 0.36	0.08 ± 0.02
	20 *μ*g/mL	92.12	0.72 ± 0.86	0.58 ± 0.43	0.14 ± 0.14
	40 *μ*g/mL	88.81	0.93 ± 0.69	0.56 ± 0.32	0.11 ± 0.10
	80 *μ*g/mL	85.24	1.54 ± 0.84	0.56 ± 0.50	0.18 ± 0.07
	160 *μ*g/mL	81.60	2.06 ± 0.94	0.50 ± 0.44	0.09 ± 0.11
	320 *μ*g/mL	74.96	2.06 ± 0.61	0.82 ± 0.35	0.02 ± 0.03

Data are significant in relation to negative control group (solvent) at ^∗∗∗^P < 0.001/one-way ANOVA with Tukey's post-test. (a) Locus nonspecific revertants (suppression by forward mutation). (b) Locus-specific revertants. (c) Mean and S.D. per five independent experiments.

## Data Availability

The data used to support the findings of this study are included within the article.
