# Corrigendum to “Biochemical and Biological Profile of Parotoid Secretion of the Amazonian* Rhinella marina* (Anura: Bufonidae)”

**DOI:** 10.1155/2019/4209743

**Published:** 2019-05-27

**Authors:** Daniel S. S. de Medeiros, Tiago B. Rego, Ana P. de A. dos Santos, Adriana S. Pontes, Leandro S. Moreira-Dill, Najla B. Matos, Juliana P. Zuliani, Rodrigo G. Stábeli, Carolina B. G. Teles, Andreimar M. Soares, Angelo R. de M. Sperotto, Dinara J. Moura, Jenifer Saffi, Cleópatra Alves da Silva Caldeira, Daniel Carvalho Pimenta, Leonardo A. Calderon

**Affiliations:** ^1^Centro de Estudos de Biomoléculas Aplicadas à Saúde (CEBio), Fiocruz-Rondônia and Universidade Federal de Rondônia, Porto Velho, 76812-245, Brazil; ^2^Plataforma de Bioensaios de Malária e Leishmaniose (PBML), FIOCRUZ-Rondônia, Porto Velho, 76812-245, Brazil; ^3^Instituto Nacional de Epidemiologia na Amazônia Ocidental, Porto Velho, 76812-245, Brazil; ^4^Laboratório de Imunologia Aplicada à Saúde, FIOCRUZ-Rondônia, Porto Velho, 76812-245, Brazil; ^5^Laboratório de Microbiologia, FIOCRUZ-Rondônia, Porto Velho, 76812-245, Brazil; ^6^Laboratório de Genética Toxicológica, Universidade Federal de Ciências da Saúde de Porto Alegre, Porto Alegre, 90050-170, Brazil; ^7^Laboratório de Bioquímica e Biofísica, Instituto Butantan, Avenida Vital Brazil, 1500, 05503-900 Sao Paulo, SP, Brazil

In the article titled “Biochemical and Biological Profile of Parotoid Secretion of the Amazonian* Rhinella marina* (Anura: Bufonidae)” [1], Drs. Cleópatra Alves da Silva Caldeira and Daniel Carvalho Pimenta, who were responsible for the mass spectrometry analysis, were missing from the authors' list. The corrected authors' list is shown above and updated in place.

## References

[1] Medeiros D. S., Rego T. B., Santos A. P. (2019). Biochemical and biological profile of parotoid secretion of the Amazonian *Rhinella marina* (Anura: Bufonidae). *BioMed Research International*.

